# Fascinating Frontier, Nanoarchitectonics, as Method for Everything in Materials Science

**DOI:** 10.3390/ma18225196

**Published:** 2025-11-15

**Authors:** Katsuhiko Ariga

**Affiliations:** 1 Research Center for Materials Nanoarchitectonics (MANA), National Institute for Materials Science (NIMS), 1-1 Namiki, Tsukuba 305-0044, Japan; ariga.katsuhiko@nims.go.jp; 2 Graduate School of Frontier Sciences, The University of Tokyo, 5-1-5 Kashiwanoha, Kashiwa 277-8561, Japan

**Keywords:** atomic and molecular level structure, biological material, living cell, nanoarchitectonics, nanomaterial, nanostructured functional material, polymeric material, supramolecular chemistry

## Abstract

Methodological fusion of materials chemistry, which enables us to create materials, with nanotechnology, which enables us to control nanostructures, could enable us to create advanced functional materials with well controlled nanostructures. Positioned as a post-nanotechnology concept, nanoarchitectonics will enable this purpose. This review paper highlights the broad scope of applications of the new concept of nanoarchitectonics, selecting and discussing recent papers that contain the term ‘nanoarchitectonics’ in their titles. Topics include controls of dopant atoms in solid electrolytes, transforming the framework of carbon materials, single-atom catalysts, nanorobots and microrobots, functional nanoparticles, nanotubular materials, 2D-organic nanosheets and MXene nanosheets, nanosheet assemblies, nitrogen-doped carbon, nanoporous and mesoporous materials, nanozymes, polymeric materials, covalent organic frameworks, vesicle structures from synthetic polymers, chirality- and topology-controlled structures, chiral helices, Langmuir monolayers, LB films, LbL assembly, nanocellulose, DNA, peptides bacterial cell components, biomimetic nanoparticles, lipid membranes of protocells, organization of living cells, and the encapsulation of living cells with exogenous substances. Not limited to these examples selected in this review article, the concept of nanoarchitectonics is applicable to diverse materials systems. Nanoarchitectonics represents a conceptual framework for creating materials at all levels and can be likened to a method for everything in materials science. Developing technology that can universally create materials with unexpected functions could represent the final frontier of materials science. Nanoarchitectonics will play a significant part in achieving this final frontier in materials science.

## 1. Introduction

Advances in information technology (IT) are transforming our lifestyles. Furthermore, remarkable advances in artificial intelligence (AI) are bringing about further social change. Although significant progress has been made in cyberspace, this is based on the development of functional materials in the real world. Today’s society faces many challenges. Furthermore, improvements to everyday life are constantly being pursued. Addressing these challenges requires the development of functional materials that can solve these issues. The active development of functional materials for a variety of applications is currently underway. Energy-related technologies, such as fuel cells [1,2,3,4,5,6], solar cells [7,8,9,10,11,12] and other energy generation technologies [13,14,15,16,17], various batteries [18,19,20,21,22] and supercapacitors [23,24,25,26,27,28], are exploring useful materials and how to structure them. This research trend is also prevalent in ecology and the environment, including the detection of specific substances [29,30,31,32,33,34], the removal of pollutants [35,36,37,38,39] and microplastics [40,41,42,43,44,45], achieving carbon neutrality [46,47,48,49,50], and the effective use of biomass [51,52,53,54,55,56]. In biomedical fields such as drug delivery [57,58,59,60,61,62], biosensing [63,64,65,66,67], virus prevention [68,69,70], cancer treatment [71,72,73] and various medical technologies [74,75,76,77,78,79,80], the development of functional materials—not just biochemical technologies—holds the key. Furthermore, developing semiconductor materials [81,82,83,84] and controlling their functions [85,86,87,88,89,90] are important for devices and sensors that support information technology. While the development of such functional materials appears to be progressing in a specific way at first glance, there is a consistent flow. It is necessary to develop materials individually to meet various targets and demands. However, it is even more important to take a step back and understand the broader trends in scientific and technological development. Such an understanding enables diverse developments to be interconnected, leading to more rational scientific and technological progress. So, where are materials science and technology headed? Rather than focusing on the development of methods or techniques for creating specific products, this review discusses a unified, comprehensive concept of materials science. To this end, this review provides an overview of developments in materials science and nanotechnology to date, introducing the concept of nanoarchitectonics as a successor to these fields. Rather than considering this concept systematically, this review demonstrates its application in many fields through showing divers applicable targets. This illustrates the broad impact that the concept can have. The purpose of this review is to explore the future of materials science comprehensively, rather than presenting anything specific.

In the early stages, humanity sought to improve life’s conveniences by procuring useful materials from nature. Through experience, they also learned the techniques to process these materials and modify them into alloys and other substances. However, this trend was fundamentally changed by advances in materials science, particularly in the 20th century. Instead of being limited to using raw materials obtained from nature, it became possible to create new materials through systematic academic research. This trend continues to this day. While efforts are being made to analyze structures [91,92,93,94] and understand phenomena [95,96,97,98,99,100,101] in fields such as physics, physical chemistry and biology, advances in materials-related chemistry that can create new materials have had a significant impact. The creation, understanding and utilization of new functional materials through organic chemistry [102,103,104,105,106], inorganic chemistry [107,108,109,110,111,112,113], coordination chemistry [114,115,116,117,118], polymer chemistry [119,120,121,122,123,124,125,126], other materials chemistry [127,128,129,130,131,132] and biochemistry [133,134,135,136,137,138,139] continues to this day. Breakthroughs in materials-related chemistry have been instrumental in advancing humanity.

Throughout this ongoing development, humanity has realized a very important fact. While the properties and functions of a material are important, its structure also plays a vital role. Even the same material can have dramatically improved or entirely new functions when its size and internal structure differ. Controlling its structure at the nanoscale is particularly effective. Nanostructures offer many advantages. When materials are manipulated at an extremely small scale, phenomena such as quantum effects [140,141,142,143,144] emerge. Nanostructured materials have a significantly larger interfacial area per unit volume, which enhances their catalytic functions [145,146]. Organizing functional units at the nanoscale enables efficient energy and electron transfer [147,148]. In other words, developing materials with more advanced functions requires controlling the material’s nanostructure as well as developing the materials themselves. The advent of nanotechnology has clearly initiated this research trend. Nanotechnology continues to evolve actively today. It is now possible to observe [149,150,151,152], manipulate [153,154,155,156] and study phenomena [157,158,159,160,161,162,163,164,165] at the atomic, molecular, and nanoscopic levels. These techniques have become indispensable for understanding materials and phenomena. It could be argued that the development of nanotechnology represents a second breakthrough in the development of functional materials.

**Figure 1:**
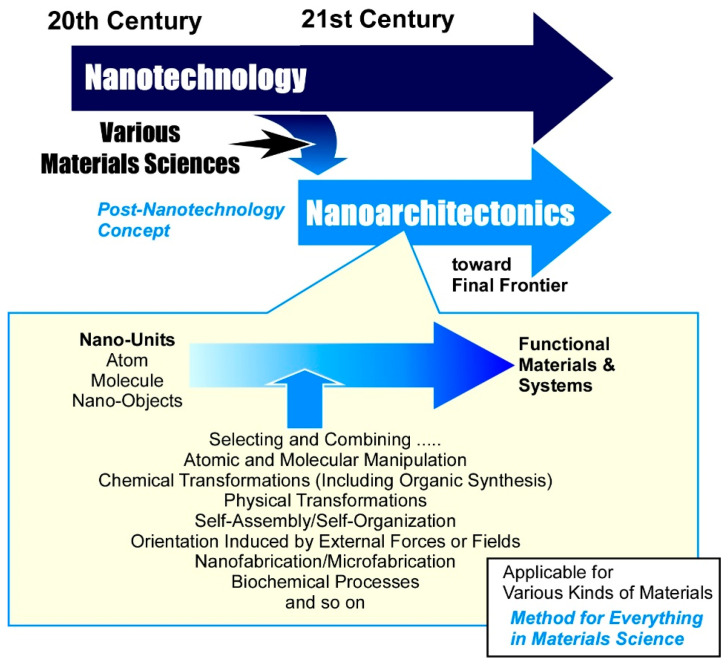
History and outline of the nanoarchitectonics concept.

The next step will require the development of an integrated concept. This could represent a significant milestone in the development of functional materials. In other words, we need to combine materials chemistry, which enables us to create materials, with nanotechnology, which enables us to control nanostructures. Positioned as a post-nanotechnology concept, nanoarchitectonics will enable this purpose (Figure 1) [166]. Just as Richard Feynman pioneered nanotechnology in the mid-20th century [167,168], Masakazu Aono proposed nanoarchitectonics during the transition from the 20th to the 21st century [169,170]. Nanoarchitectonics integrates nanotechnology and materials chemistry to build functional material systems from basic elements such as atoms, molecules and nanomaterials [171]. Nanoarchitectonics is not an entirely new concept. Rather, it seeks to integrate existing concepts and create a stream of clear breakthroughs. Methods of assembling atoms and molecules to create functional structures have been realized through self-assembly in supramolecular chemistry and related fields such as molecular/materials assembly science [172,173,174,175,176], metal–organic frameworks (MOFs) [177,178,179,180,181,182,183] in coordination chemistry, and covalent organic frameworks (COFs) [184,185,186,187,188], which have been cleverly developed from polymer chemistry. Mesoporous and related materials [189,190,191,192,193] have also been created by materials chemistry using molecular assemblies as templates. In interface science, the construction of functional thin films from molecules and materials has been widely demonstrated using methods such as self-assembled monolayers (SAMs) [194,195,196,197,198,199], the Langmuir–Blodgett (LB) method [200,201,202,203,204] and layer-by-layer (LbL) assembly [205,206,207,208,209]. However, this concept has developed rather independently and has not become a unified driving force in research. The importance of creating an integrated concept, even if it is not entirely novel, is evident from the success of nanotechnology. Although there was a need for research to elucidate nano-level phenomena, it was nanotechnology that became a symbolic unifying concept, leading to the creation of a major research trend. Nanoarchitectonics is now taking on this role.

Nanoarchitectonics involves building functional materials using existing knowledge and technologies. This process involves creating materials that incorporate a variety of technological elements. Building functional material systems from atoms, molecules, and nanomaterials can incorporate a variety of technologies and sciences. These materials can be created by selecting and combining atomic and molecular manipulation, chemical transformations (including organic synthesis), physical transformations, self-assembly/self-organization, orientation induced by external forces or fields, nanofabrication/microfabrication and biochemical processes [210]. Unlike conventional supramolecular methods such as self-assembly, MOF and COF, the nanoarchitectonics processes are supposed to comprehensively integrate several methods together even including physical fabrication and biological treatments. These integrated material fabrication methods also offer advantages in terms of structural fabrication. Compared to single processes or processes based on a single equilibrium, combining multiple processes offers significant advantages when it comes to building asymmetric and hierarchical structures [211]. However, nanoscale interactions can sometimes involve uncertainty, including contributions from thermal fluctuations and quantum effects [212]. The inputs added to functional materials do not necessarily equate to the sum of their components as they interfere with each other. Therefore, rather than being additive, the effect is that of a harmonized whole [213]. This is very similar to the functional systems of living organisms, in which multiple functional units link together and work collaboratively within thermal fluctuations. This is a common characteristic of nanoarchitectonics. Conversely, one could argue that the ultimate goal of nanoarchitectonics is to build highly functional systems similar to those found in living organisms [214].

The principles and characteristics of nanoarchitectonics are general and universal, and are independent of the materials used, their functions, and their applications. In recent years, the number of papers claiming to be in this field has increased. The range of papers featuring the term ‘nanoarchitectonics’ in their titles is diverse. Many focus on basic science, such as the study of chemical materials [215,216,217,218,219] and structural control [220,221,222,223], the exploration of physical phenomena [224,225], and fundamental biochemical approaches [226,227,228,229,230]. However, nanoarchitectonics is also widely used in application-oriented fields, including solar cells [231,232,233,234], fuel cells [235,236,237,238,239], batteries [240,241,242,243], supercapacitors [244,245,246,247,248,249] and other energy applications [250,251,252,253,254], environmental issues [255,256,257,258], catalysis [259,260,261,262,263,264], devices [265,266,267,268,269], sensors [270,271,272,273], biosensors [274,275,276,277], drug delivery [278,279,280,281,282] and biomedical applications [283,284,285,286,287,288]. As all materials are essentially composed of atoms and molecules, the nanoarchitectonics methodology, which is built from these components, may be applicable to all materials. If the ultimate goal of physics is to establish a theory of everything [289], nanoarchitectonics could be considered a method for everything in materials science [290,291]. Nanoarchitectonics is also the final frontier in the quest to assemble highly functional systems akin to those found in living organisms. The challenge of nanoarchitectonics is to achieve, within our own lifetimes, a feat equivalent to the creation of highly functional systems from atoms and molecules, which evolution in nature has taken billions of years to achieve.

To this end, this review paper will introduce the concept of nanoarchitectonics by highlighting recent papers that include the term in their title. Finally, future directions will be discussed in reference to these examples. Next, this paper will discuss the potential of nanoarchitectonics as a method for a wide range of applications. Its perspective section will also consider what is required for nanoarchitectonics to become the ultimate frontier in the development of functional materials.

## 2. Research Target in Nanoarchitectonics from Atom to Living Cell

To demonstrate the widespread use of the concept of nanoarchitectonics, this section presents a number of examples of research papers. To illustrate its deep involvement in research, only papers containing the term ‘nanoarchitectonics’ in the title have been selected. Many of the examples are from recent papers, showing that nanoarchitectonics is used in popular research fields. Furthermore, to demonstrate the wide range of research content targeted, the papers have been selected to show divergence rather than systematic organization. Because these examples have various functions with certain integration, the examples are basically represented in the order of system sizes from atoms and molecules to materials and living systems. One strict selection criterion is that these research examples have the term of nanoarchitectonics in their paper title. Because of rapid growth of nanoarchitectonics research in these days, the most of them are selected from those published in these few years.

### 2.1. Nanoarchitectonics for Dopant Atom

In their paper, ‘Nanoarchitectonics for controlling the number of dopant atoms in solid electrolyte nanodots’, Hasegawa et al. reported a method for controlling the number of dopant atoms in solid electrolytes [292]. This represents a nanoarchitectonics strategy for achieving discrete electrical properties. Controlling the movement of electrons and holes is a key challenge in today’s highly information-driven society. The ultimate technology in solid-state nanoionics, aimed at applications in energy storage, sensing and brain-based information processing, is the ability to control material properties at the atomic scale. The proposed study uses α-Ag_2_S nanodots containing non-stoichiometric excesses of Ag^+^ ions and electrons as a model system. The dopant can be controlled and manipulated in discrete steps by changing the electrochemical potential. In thermodynamic equilibrium, the Gibbs free energy of solid electrolyte materials can be minimized through the formation of point defects, i.e., Frenkel or Schottky equilibrium. The number of defects increases when one of the components is non-stoichiometric. This significantly affects the electronic and/or ionic conductivity of the material. In particular, as approaching to the nanoscale, the equilibrium defect concentration can increase by orders of magnitude due to the increased entropy of the system. By limiting the size of nanodots, the number of nonstoichiometric defects that function as dopants and undergo electrochemical transformation can be tailored. This approach proposed a strategy for controlling the number of dopant atoms in α-Ag_2+δ_S nanodot solid electrolytes on platinized silicon wafer substrates by adjusting the electrochemical potential. Limiting the number of non-stoichiometric dopants increases the distance between adjacent electrochemical potential levels, enabling the precipitation of atoms. This approach is an attractive paradigm in nanoarchitectonics for developing nanodevices based on single-ion/single-atom transfer.

### 2.2. Nanoarchitectonics for Pentagon Defect in Carbon

**Figure 2:**
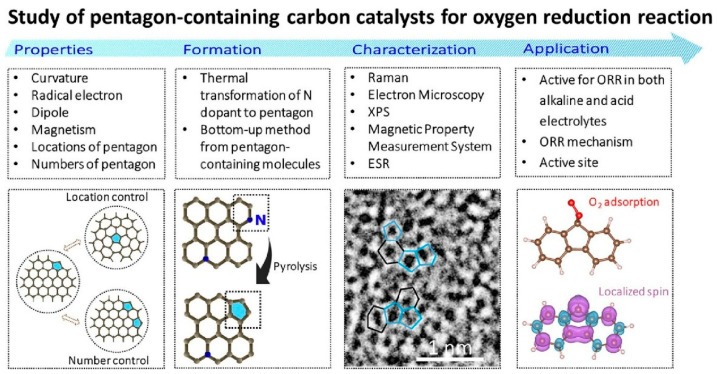
Summary of studies of pentagon-containing carbon catalysts for oxygen reduction reaction (ORR). Reproduced under terms of the CC-BY license [294]. Copyright 2025 Wiley-VCH.

Research into nanoarchitectonics, which involves manipulating the carbon atom framework in carbon materials at the atomic level, has also been reported. Chen et al. recently reported a method for controlling the oxygen reduction reaction (ORR) by manipulating pentagon structures in carbon materials [293]. In their review paper, ‘Nanoarchitectonics for pentagon defects in carbon: properties and catalytic role in oxygen reduction reaction’, Chen et al. provide a comprehensive summary of the formation mechanism, characterization, spin, oxygen adsorption and ORR catalytic activity of carbon catalysts containing pentagon defects [294]. Carbon materials have attracted considerable attention as a sustainable and economical alternative to noble metal catalysts. In recent years, it has been reported that introducing pentagon structures into graphitic carbon promotes ORR catalytic activity. As illustrated in Figure 2, this review explores the formation mechanism of pentagon defects in carbon materials, from high-temperature annealing to bottom-up synthesis strategies. Notably, carbon catalysts containing pentagons exhibit high catalytic activity even under acidic conditions and often outperform *N*-doped carbon catalysts. These catalysts are highly durable and approach the ORR performance of Pt-based catalysts. This high activity is attributed to the unique electronic structure induced by the pentagonal defects which lead to spin formation. Pentagonal structures have been introduced into carbon frameworks using various nanoarchitectonics methods, including high-temperature treatment, bottom-up synthesis and selective dopant removal. Furthermore, considerable attention has also been given to the synergistic effect of heteroatom dopants, such as nitrogen and sulphur, with pentagonal defects.

### 2.3. Single-Atom Nanoarchitectonics for Oxygen Evolution Reaction

**Figure 3:**
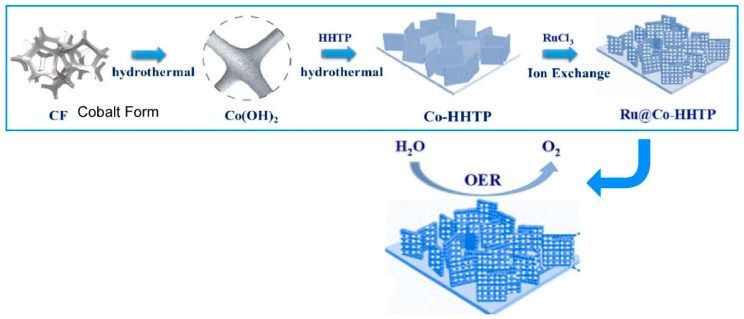
Introduction of atomically dispersed Ru into Co-HHTP (HHTP = 2,3,6,7,10,11-hexahydroxytriphenylene) for excellent oxygen evolution reaction (OER) performance. Reprinted with permission from [295]. Copyright 2024 American Chemical Society.

The design of single-atom catalysts is also being explored in nanoarchitectonics. One of the key targets for catalysis is the development of electro-catalysts for the oxygen evolution reaction (OER), which are essential for producing green hydrogen through water electrolysis but also present a significant research challenge. In their paper, ‘Ru single-atom nanoarchitectonics on Co-based conducting metal–organic frameworks for enhanced oxygen evolution reaction’, Du, Xu and coworkers prepared ruthenium (Ru) single-atom-decorated Co-HHTP (HHTP = 2,3,6,7,10,11-hexahydroxytriphenylene) (Ru@Co-HHTP) using solvothermal and ion-exchange methods (Figure 3) [295]. First, Co(OH)_2_ was grown on a substrate using a solvothermal method and then converted to Co-HHTP by adding HHTP during the solvothermal process. Furthermore, a single Ru atom was immobilized on the Co-HHTP using an ion-exchange method. This study aims to demonstrate that introducing atomically dispersed Ru into Co-HHTP increases the electrochemically active area, improves charge transfer capability and optimizes the electronic structure of Co-HHTP. Systematic experiments suggest that atomically dispersed Ru can optimize the electronic structure and electronic conductivity of Co-HHTP, resulting in low overpotential, a small Tafel slope, a large electrochemically active surface area, an excellent charge transfer capability and a strong electronic interaction between Co and Ru. Consequently, excellent OER performance was demonstrated. Furthermore, this is expected to greatly accelerate the development of alkaline water splitting for practical applications.

### 2.4. Single-Atom Nanoarchitectonics for Robotics

**Figure 4:**
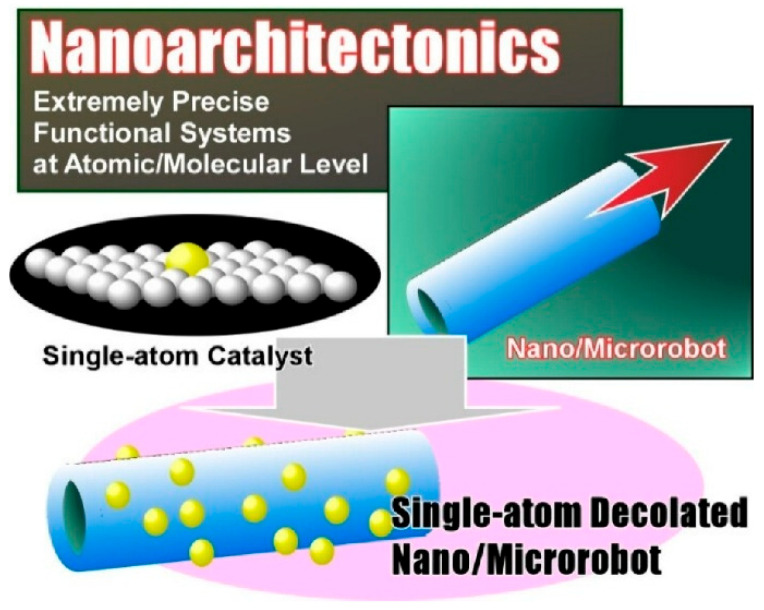
Basic concept for single-atom nanoarchitectonics for robotics and other functions. Reproduced under terms of the CC-BY license [296]. Copyright 2025 American Chemical Society.

The development of nanoarchitectonics, which involves creating objects that function like nanorobots and microrobots, is also underway. The ultimate goal of this field of research is to construct highly precise, functional, dynamic systems originating at the atomic and molecular level. In such robotic structures, single-atom catalysts act as active components that power the robots. In their review paper, ‘Single-atom nanoarchitectonics for robotics and other functions’, Jancik-Prochazkova et al. discuss functional systems implemented with single-atom catalysts as active elements in the field of nano/micro-robotics (Figure 4) [296]. Single-atom-modified nano/microrobots are dynamic systems that utilize the catalytic activity of single atoms and can enhance propulsion or provide catalytic functionality. The review paper covers three key topics: research trends in single-atom catalysts, single-atom-modified nano/microrobots and additional applications of single-atom nanoarchitectonics. It also considers the future development and direction of single-atom-modified nano/microrobots. It is particularly expected that the next generation of intelligent single-atom-modified nano/micro-robots will be developed using artificial intelligence (AI).

### 2.5. Nanoarchitectonics of Nanoparticle

**Figure 5:**
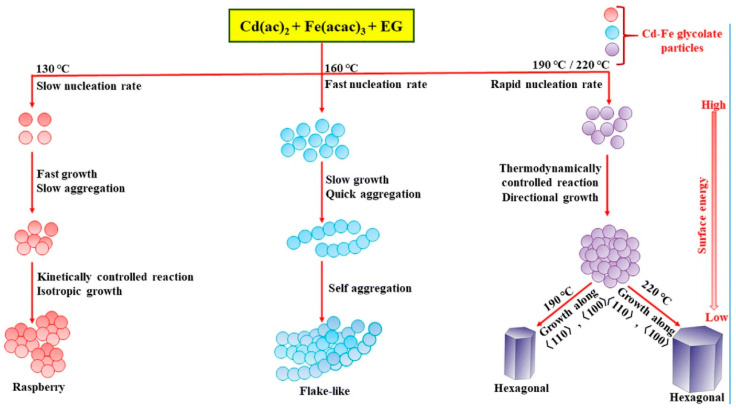
Nanoarchitectonics of CdFe_2_O_4_ nanoparticles with different through varying the reaction conditions where nucleation and growth are key factors affecting the morphology of Cd–Fe glycolate. Reprinted with permission from [297]. Copyright 2024 Royal Society of Chemistry.

Functional nanoparticles and related nanostructures are important targets in the field of nanoarchitectonics. In their paper, ‘Nanoarchitectonics of CdFe_2_O_4_ nanoparticles with different morphologies synthesized using a thermal decomposition approach and studies of their peroxidase-like activity’, Gangwar and Jeevanandam reported that CdFe_2_O_4_ nanoparticles with different morphologies can be synthesized using a simple thermal decomposition method with Cd–Fe glycolate as a precursor [297]. Varying the reaction conditions resulted in CdFe_2_O_4_ nanoparticles with various morphologies, such as flake, raspberry and hexagonal (Figure 5). Nucleation and growth are key factors affecting the morphology of Cd–Fe glycolate. The synthesis temperature influences these factors, and nanoparticles of various shapes are formed through kinetic control and surface energy reduction. Furthermore, CdFe_2_O_4_ nanoparticles exhibit morphology-dependent magnetic properties. Furthermore, CdFe_2_O_4_ nanoparticles exhibit peroxidase-like activity in the presence of hydrogen peroxide. The catalytic activity of CdFe_2_O_4_ nanoparticles is affected by their morphology, particle size, surface area and Fe^3+^ ion concentration. Their catalytic activity is superior to that of the natural enzyme horseradish peroxidase (HRP). CdFe_2_O_4_ nanoparticles may be useful in applications such as magnetic devices, catalysis and sensing.

### 2.6. Tubular Nanoarchitectonics

**Figure 6:**
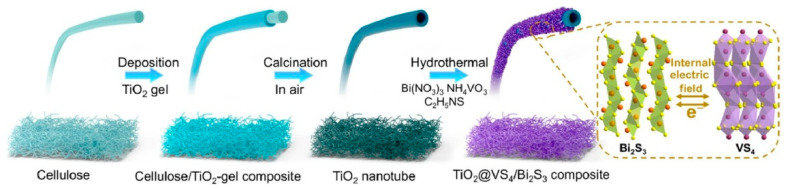
Tubular nanoarchitectonics of titania@vanadium-sulfide/bismuth-sulfide composite for effective sodium-ion storage, in which cellulose-derived titania nanotubes were employed as a structural scaffold for synthesizing this material using sol–gel and hydrothermal methods. Reprinted with permission from [298]. Copyright 2025 American Chemical Society.

Research on nanoarchitectonics of tubular materials has also been reported. Vanadium sulfide has attracted attention as a potential anode material for sodium-ion batteries. However, its electrochemical performance is limited by poor structural stability during charge–discharge processes and a slow ion diffusion rate. In a paper titled ‘Tubular nanoarchitectonics of titania@vanadium–sulfide/bismuth–sulfide composite for effective sodium-ion storage’, Huang et al. developed a material in which VS_4_/Bi_2_S_3_ nanorods were immobilized on the surface of titania nanotubes (Figure 6) [298]. Cellulose-derived titania nanotubes were employed as a structural scaffold for synthesizing this material using sol–gel and hydrothermal methods. A metal sulfide-based double-tubular nanostructure (TiO_2_@VS_4_/Bi_2_S_3_ composite) was then constructed on this scaffold. This composite consists of VS_4_/Bi_2_S_3_ nanorods grown on the surface of the titania tubes, forming a heterogeneous interface with abundant voids and mesopores. The composite’s excellent sodium storage properties are attributed to its abundant phase interfaces and cross-linked tubular structure. The cross-linked TiO_2_@VS_4_/Bi_2_S_3_ nanotube composite effectively improves the kinetics of charge transfer and sodium ion transport and alleviates the problems of volume expansion and voltage drop. Given the common challenges involved, this study may offer valuable insights into designing heterogeneous nanocomposites with abundant phase interfaces.

### 2.7. Nanoarchitectonics of Stacked Macrocycle Nanosheet

**Figure 7:**
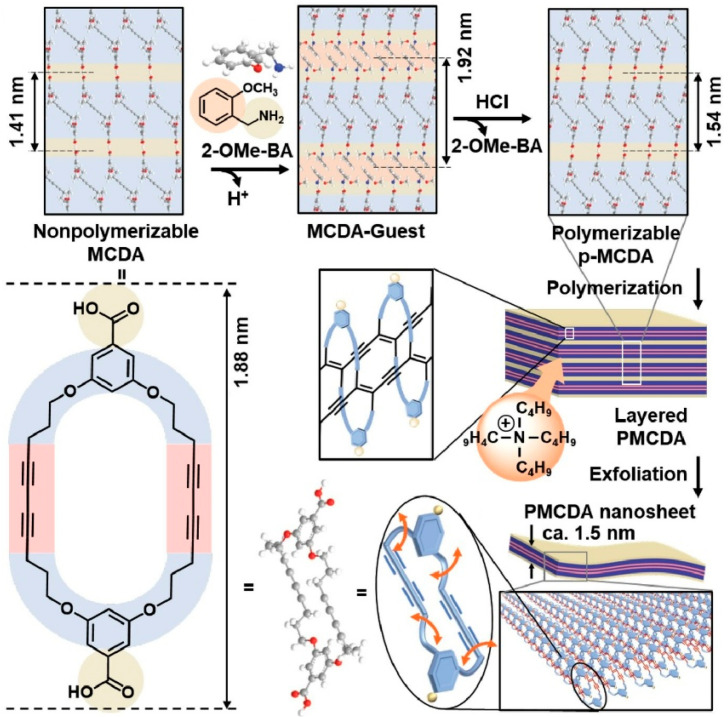
Nanoarchitectonics for exfoliation nanosheets of macrocyclic diacetylenes through self-assembly of designed macrocyclic di-acetylene monomers with chair conformations and topochemical polymerization of the diacetylene moieties. Reproduced under terms of the CC-BY license [299]. Copyright 2023 Wiley-VCH.

Due to their anisotropic ultra-thinness, 2D nanostructures consisting of monolayers or a few layers exhibit unique properties. Therefore, stacked nanosheet structures are also interesting targets for nanoarchitectonics. In their study, ‘Nanoarchitectonics of exfoliated flexible nanosheet based on laterally stacked macrocycles’, Kim, Oaki and coworkers developed a new type of exfoliated organic 2D material, as shown in Figure 7 [299]. This design demonstrates nanoarchitectonics involving the synthesis, controlled assembly, polymerization and exfoliation of macrocyclic diacetylenes. The diversity of molecular motions and conformations of the macrocycles provides potential structural flexibility for the nanosheets. Therefore, similar flexible organic 2D materials can be obtained by exfoliation by designing macrocycles containing diverse functional molecular units, resulting in the formation of exfoliable layered structures. Specifically, the self-assembly of designed macrocyclic diacetylene monomers with chair conformations leads to the formation of stacked structures containing vertically aligned, macrocycle-based layers. The exfoliable layered structure is obtained by topochemical polymerization of the diacetylene moieties. Intercalation and subsequent swelling yield nanosheets based on laterally stacked macrocycles in the aqueous phase. This design is expected to lead to soft materials such as liquid crystals, gels and composites with flexible structures and dynamic functions.

### 2.8. Nanoarchitectonics of Organic Nanosheet

Research into nanosheets that exhibit optical functions is also being conducted within the field of nanoarchitectonics. High-mobility, ultrathin, 2D organic nanosheets that are only a few molecular layers thick have attracted considerable attention. However, it is not necessarily easy to fabricate ultrathin 2D organic nanosheets that simultaneously possess high luminescence efficiency and flexibility. In a study titled ‘Hierarchical nanoarchitectonics of ultrathin 2D organic nanosheets for aqueous processed electroluminescent devices’, Zhang, Xie and coworkers demonstrated a method for fabricating uniformly sized, ultrathin 2D organic nanosheets through multifunctional supramolecular design using a solution-based technique [300]. Specifically, they incorporated methoxyl and diphenylamine groups into 3D spiro-fluorene–xanthene building blocks to successfully prepare ultrathin 2D organic nanosheets with a thickness of 19 nm through denser molecular packing, with π-π stacking and CH···π interactions supporting antiparallel and interpenetrating molecular packing in dimeric aggregates to favor the formation of ultrathin 2D organic nanosheets. Large-area, flexible 2D organic nanosheet films were fabricated by a simple drop-coating method using an aqueous nanosheet ink. Trimeric aggregates with a 2D brickwork structure were formed, restricting conformational vibration and rotation, and minimizing nonradiative deactivation in the solid state. Due to the H-aggregation mode, the resulting ultrathin 2D organic nanosheets exhibit stable blue emission and superior photoluminescence quantum yields compared to amorphous films. The crystalline OLED device performance of the 2D organic nanosheet films was achieved. These ultrathin 2D organic nanosheets could also be useful in the development of flexible, electrically pumped lasers and intelligent quantum tunnelling systems.

### 2.9. Iron N-Doped Carbon Nanoarchitectonics

The use of nanoarchitectonics in materials development has been widely studied. Nanoarchitectonics for functional carbon materials is a subject of widespread interest. In their study, ‘Iron N-doped carbon nanoarchitectonics for C-H bond activation of methylarenes and esterification reactions’, Gawande et al. reported a method for preparing scalable iron nanoparticles on nitrogen-doped carbon via wet chemical reactions followed by high-temperature pyrolysis [301]. N-doped carbon possesses electron transfer and conductive properties. In particular, its pyridinic-N and graphitic-N compositions make it efficient for synthesizing advanced metal nanomaterials that are useful for organic transformations. The resulting carbon material significantly activates O_2_ at room temperature to generate superoxide species. Introducing nitrogen dopants improves the dispersibility and stability of Fe nanoparticles and promotes C-H bond activation through the interaction between iron species and nitrogen-containing functional groups. These nitrogen-coordinated iron nanoparticles play an important role as active sites, promoting both toluene oxidation and esterification reactions. For instance, when this catalyst is used alongside *N*-hydroxyphthalimide, methylarenes can be converted into the corresponding arylaldehydes with 99% conversion and selectivity at room temperature, without the oxidation of benzaldehyde to benzoic acid occurring. Iron nanoparticles decorated with nitrogen-doped carbon catalysts could provide durable, easily recoverable and environmentally friendly metal-based catalysts.

### 2.10. Nanoarchitectonics of Ordered Mesoporous C_60_–BCN

**Figure 8:**
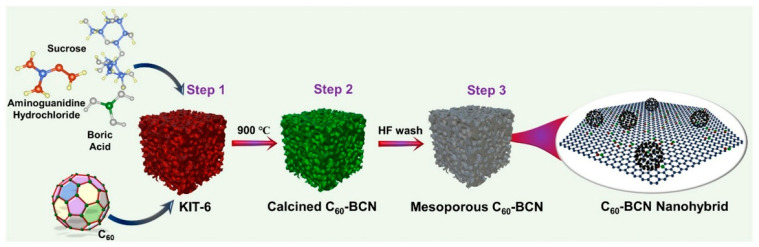
Nanoarchitectonics of high-surface-area ordered mesoporous hybrids of fullerene and borocarbon nitride (BCN) using KIT-6, a mesoporous silica with a 3D cage-type porous structure, as a hard template. Reproduced under terms of the CC-BY license [302]. Copyright 2024 Elsevier.

Nanoporous and mesoporous materials, which enable the creation of controlled pore structures, are also promising areas of research in nanoarchitectonics. Mesoporous materials, with their unique pore structure and high surface area, hold great potential for energy storage applications. They have attracted considerable interest as high-performance electrode materials for next-generation energy storage devices. In the paper ‘Hybrid nanoarchitectonics of ordered mesoporous C_60_–BCN with high surface area for supercapacitors and lithium-ion batteries’, Vinu et al. synthesized high-surface-area ordered mesoporous hybrids of fullerene and borocarbon nitride (BCN) using KIT-6, a mesoporous silica with a 3D cage-type porous structure, as a hard template (Figure 8) [302]. The resulting materials exhibit a high surface area and a uniform distribution of pores, thanks to the C_60_ nanostructures decorating the hybrids. This structural feature provides high ionic charge transport and improved electrochemical stability. Consequently, this nanoporous material demonstrated exceptional performance in energy storage. These materials are considered a unique platform for developing highly stable anode materials for energy storage devices. Various advanced spectroscopic tools can be used to analyze the presence of C_60_, and applications in novel energy storage systems such as Li^+^/Na^+^ hybrid capacitors and Na^+^/K^+^ batteries can be explored.

### 2.11. Nanoarchitectonics: MXene/Covalent Organic Framework

**Figure 9:**
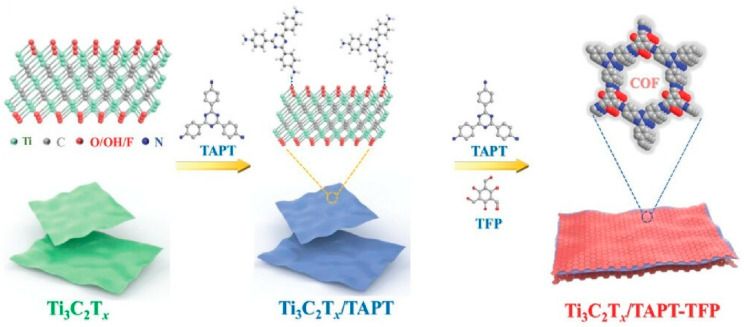
Inorganic–organic nanoarchitectonics strategy to synthesize an MXene/COF hetero-structure (Ti_3_C_2_Tx/TAPT-TFP) by assembling β-ketoenamine-linked COFs onto Ti_3_C_2_Tx MXene nanosheets. Reproduced under terms of the CC-BY license [303]. Copyright 2024 Wiley-VCH.

Research into nanoarchitectonics is also being conducted using notable materials, such as MXene nanosheets and covalent organic frameworks (COFs), as components. COFs have excellent adsorbent properties and potential. In order to utilize their capabilities, it is necessary to optimize the stacking/aggregation structure during the synthesis process. In the paper ‘Inorganic–organic nanoarchitectonics: MXene/covalent organic framework heterostructure for superior microextraction’, Zhang, Xu, Yamauchi and coworkers developed an inorganic–organic nanoarchitectonics strategy to synthesize an MXene/COF heterostructure (Ti_3_C_2_T_x/TAPT-TFP) by assembling β-ketoenamine-linked COFs onto Ti_3_C_2_Tx MXene nanosheets (Figure 9) [303]. This material is constructed as follows: First, Ti3C2Tx MXene was ultrasonically dispersed in ethanol under nitrogen bubbling. Subsequently, 4,4′,4′’-(1,3,5-triazine-2,4,6-triyl)trianiline (TAPT) monomer was added and allowed to penetrate between the MXene nanosheet layers. Following the covalent linking of 1,3,5-triformylphloroglucinol (TFP) and the TAPT monomer via a Schiff base condensation reaction, the TAPT-TFP-COF nanostructures assembled on the Ti_3_C_2_T_x_/TAPT surface to form the Ti_3_C_2_T_x_/TAPT-TFP heterostructure. This material retains the 2D structure and high adsorption capacity of MXene, as well as the large specific surface area and hierarchical porous structure of COF. It also provides a method for synthesizing COF-MXene hybrid materials, which are useful for micro-extracting environmental pollutants. It also enables the extraction of trace organochlorine in fruit and vegetable samples. These materials have been found to exhibit low detection limits, wide linearity ranges and acceptable reproducibility in pesticide analysis. These nanoarchitectonics approaches offer opportunities to address challenges in COF nanoengineering through organic–inorganic hybridization strategies.

### 2.12. Covalent Nanoarchitectonics: Polymer Synthesis

Nanoarchitectonics involves designing molecular and material structures through intermolecular interactions, such as hydrogen bonding and electrostatic interactions, as well as covalent bonds. The synthesis of polymers and related materials is also included in nanoarchitectonics research. Polymer synthesis, which involves linking monomer units, is also considered a nanoarchitectonics process. Indeed, biological systems utilize this strategy adeptly, producing biopolymers with perfectly defined sequences and structures. In the review paper, ‘Covalent nanoarchitectonics: polymer synthesis with designer structures and sequences’, Matsumoto et al. introduced notable examples of polymer synthesis with controlled sequences and structures from the past and present, including one-dimensional sequential polymers, multidimensional polymers based on supramolecular template polymerization and crystal/liquid crystal-based polymerization, and covalent organic frameworks [304]. If these technological and theoretical strategies are realized, artificial polymers will likely exhibit functionality comparable to that of biological polymers. In particular, the ability to easily control the atomic arrangement of polymers would dramatically expand the available materials library. From a practical viewpoint, polymers play an essential role in materials technology, so the development of precisely controlled polymers using a nanoarchitectonics approach would be a significant achievement.

### 2.13. Polymer Nanoarchitectonics for Synthetic Vesicle

In addition to synthesizing polymers, controlling their assembly structure is also part of nanoarchitectonics research. In some cases, this process can mimic biological structures. In a study titled ‘Polymer nanoarchitectonics for synthetic vesicles with human erythrocyte-like morphology transformation’, Yoshida demonstrated that synthetic polymer vesicles undergo morphological transformations resembling human red blood cells in response to temperature changes [305]. The photopolymerization-induced self-assembly of poly(methacrylic acid)-block-poly(n-butyl methacrylate-random-methacrylic acid) in a 70% methanol aqueous solution produced dimpled spherical vesicles that resemble red blood cells. These polymer vesicles exhibited transformation behaviors similar to those of red blood cells under various conditions. For instance, they transformed into sawtooth or cup-like shapes, resembling red blood cells. The transformations of both the vesicles and red blood cells were reversible and repeatable. However, repeated cycles resulted in irreversible deformations. The pathways of transformation of the vesicles were also similar to those of red blood cells. Furthermore, synthetic polymer vesicles underwent morphological changes resembling human red blood cells in response to temperature changes. When heated in solution, spherical, red blood cell-like vesicles transformed into echinocyte-like, sawtooth-shaped vesicles. This occurred as the copolymer constituents were released from the surface of the vesicles and expanded. As the concentration of the vesicles increased, they transformed into cup-shaped vesicles resembling red blood cells. These observations suggest that synthetic polymer vesicles could facilitate a deeper understanding of the unique properties of red blood cell membranes at the molecular level. Nanoarchitectonics may elucidate the similarities between living cells and non-living structures at the molecular level. This suggests that non-natural polymer vesicles could significantly contribute to our understanding of the causes and mechanisms of intractable diseases, and to their treatment.

### 2.14. Nanoarchitectonics in Colloidal Hydrogel

The concept of nanoarchitectonics is also driving the development of advanced colloidal hydrogels with enhanced functionality for a wide range of applications. In the review paper titled ‘Nanoarchitectonics in colloidal hydrogels: Design and applications in the environmental and biomedical fields’, Kim, Kumar and coworkers discuss the technological landscape of nanoarchitectonics with a focus on colloidal hydrogels [306]. In particular, integrating nanoparticles and polymers enables the synthesis of multifunctional nanogel platforms through the chemical or physical crosslinking of nanoparticles, polymers, and small molecules. The combined effects of the nanoparticles’ composition, size, shape, structure, binding mechanism, and molecular crosslinker properties largely determine these synergistic properties. Colloidal hydrogels have great potential for use in biomedical applications such as environmental remediation, drug delivery, wound dressings and therapeutic diagnostics. Nanoarchitectonics in colloidal hydrogels plays an increasingly important role in providing innovative solutions to pressing global challenges in healthcare and environmental sustainability. In advanced applications, nanocomposite hydrogel nano-units provide bioelectrical interfaces for signal generation and transduction, enabling the creation of real-time sensing materials.

### 2.15. Langmuir Nanoarchitectonics

**Figure 10:**
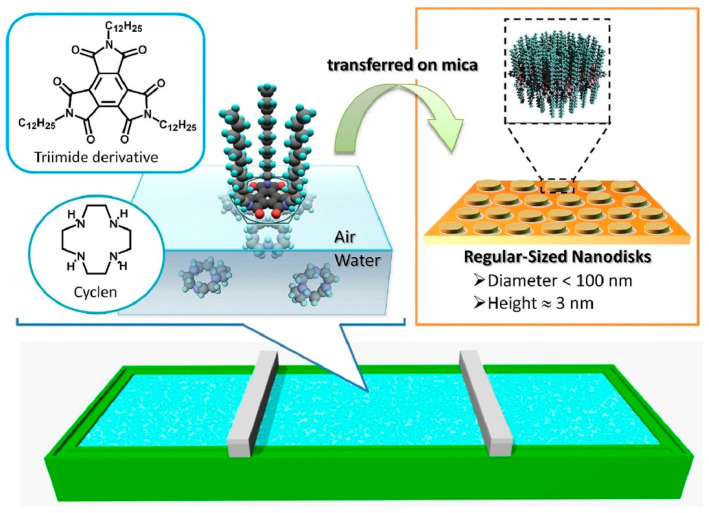
Nanoarchitectonics methodology for producing monodisperse, regularly sized disks with thicknesses of several nanometers and diameters of less than 100 nm, using Langmuir monolayers as the fabrication medium. Reprinted with permission from [307]. Copyright 2012 American Chemical Society.

Research into interfacial processes with the nanoarchitectonics concept has made significant contributions. In the study, ‘Langmuir nanoarchitectonics: one-touch fabrication of regularly sized nano-disks at the air–water interface’, Mori et al. presented a methodology for producing monodisperse, regularly sized disks with thicknesses of several nanometers and diameters of less than 100 nm, using a Langmuir monolayer as the fabrication medium (Figure 10) [307]. In this method, a monolayer of the amphiphilic triimide tri-n-dodecylmellitic acid triimide is formed on an aqueous phase containing the water-soluble macrocyclic oligoamine 1,4,7,10-tetraazacyclododecane (cyclene). In this combination, the imide moiety acts as a hydrogen bond acceptor and interacts weakly with the secondary amine moiety of cyclene, which acts as a hydrogen bond donor. Using the Langmuir–Schaefer method, Langmuir monolayers were transferred onto mica to form disk-like structures with heights of approximately 3 nm and tunable diameters in the range of tens of nanometers. The use of weak interactions allows for more precise control of domain size in two dimensions. The organic assemblies are transferred to metallic nanostructures via sputter deposition of metal onto the LB film. The size distribution of the disk-like objects is sufficiently narrow to be within the quantum disk dimension. This enables the fabrication of two-dimensional nanostructures with regular shapes, such as quantum disks. Controlled fabrication of well-defined nanostructures through a simple macroscopic process that can be carried out at room temperature provides a unique approach to economical, energy-efficient nanofabrication.

### 2.16. Layer-by-Layer Nanoarchitectonics

Layer-by-layer (LbL) assembly is a versatile interfacial process and a promising approach for nanoarchitectonics research. In the study titled ‘Tannic acid facilitated layer-by-layer nanoarchitectonics for hydrophobic conductive cotton fabric with improved stability for thermal management and flexible sensing’, Zeng et al. fabricated conductive cotton fabrics by layering tannic acid and cellulose nanofibre-dispersed carbon nanotubes onto the fabric surface using the LbL method [308]. First, clean cotton fabrics were alternately immersed in a tannic acid solution and a cellulose nanofiber-dispersed carbon nanotube dispersion to allow self-assembly. Next, the fabrics were immersed in an ethanol solution of stearic acid to enhance hydrophobicity. Tannic acid and carbon nanotubes exhibit π-π interactions, while cellulose nanofibers and tannic acid exhibit hydrogen-bonding interactions. Therefore, tannic acid firmly integrates the cellulose nanofiber-dispersed carbon nanotubes into the cotton fabrics. Furthermore, the hydrogen-bonding interaction between tannic acid and stearic acid contributes to the coating of stearic acid on the fibers. The strong adhesive force of tannic acid fixed the cellulose nanofiber-dispersed carbon nanotubes firmly to the fabric surface. Meanwhile, the stearic acid enhanced the stability of the conductive cotton fabrics, providing excellent resistance to tape peeling, ultrasonic cleaning and water droplet impact. The hydrophobic, conductive cotton fabrics exhibited excellent electrothermal conversion capabilities and stable thermal performance. Furthermore, they exhibited high sensitivity, a fast response time and excellent sensing stability in sensor applications, effectively monitoring human body movements such as joint bending, chewing and swallowing. These properties make them promising candidates for energy-efficient heating textiles and temperature-regulating smart wearable devices. This flexible sensor has excellent breathability and sensing performance, enabling accurate monitoring of physiological activities in patients. It has great potential for monitoring human health and providing personalized medical diagnoses.

### 2.17. Chiral and Topological Nanoarchitectonics

Not only does fabricating nanoscale structures with specific topologies confer unique properties on functional materials, but it also has the potential to elucidate the underlying functional mechanisms of many natural systems. The topic of nanoarchitectonics, with a focus on chirality and topology, is fascinating. In the review paper, ‘Emergent chiral and topological nanoarchitectonics in self-assembled supramolecular systems’, Liu and coworkers outline the progress made in constructing emergent chiral and topological nanoarchitectonics using self-assembly methods [309]. The authors focus particularly on toroids, catenanes, Möbius strips, spirals and fractals. The design of building blocks and various self-assembly strategies towards these target structures are also highlighted, outlining feasible approaches to facilitate the tailor-made construction of mesoscopic structures. The integration of chirality into emergent chiral and topological nanoarchitectures in self-assembled supramolecular systems can be achieved through strategies such as symmetry breaking, topological chirality or the use of enantiopure components. While these studies demonstrate great potential, there are significant knowledge gaps in this cutting-edge field and further demonstration of functionality and practical applications is essential. Particular attention should be paid to research closely related to chirality and its unique topological configurations in fields such as mechanical, optical, optoelectronic and magnetic materials.

### 2.18. Nanozyme Nanoarchitectonics

Nanozymes that mimic the functions of enzymes are also a focus of nanoarchitectonics research. In particular, two-dimensional (2D) nanozymes exhibit properties that mimic enzymes such as peroxidase, oxidase, catalase, and superoxide dismutase. These 2D nano-systems are in increasing demand due to their ability to enable ultrasensitive, label-free detection, real-time analysis, point-of-care testing and multiplexed biomarker detection. In the review article titled ‘Two-dimensional nanozyme nanoarchitectonics customized electrochemical bio diagnostics and lab-on-chip devices for biomarker detection’, Chang et al. discuss the advantages of 2D nanozymes and recent advances in their applications in biosensing [310]. The development of 2D nanozymes with diverse functions offers great potential for novel nanozyme-based diagnostic methods and biosensors. The article reviews the current status of 2D nanozymes, focusing on their synthesis, biocatalytic activity, and advances in the development of bio-detection and lab-on-chip devices for cancer and non-cancer biomarker detection. Further detailed research is required to facilitate the application of 2D nanozymes in clinical diagnostics. Notably, integrating wearable devices for personalized health monitoring and measuring glucose and albumin biomarkers with IoT/IoMT and AI/ML could provide an attractive solution for automated interpretation of diagnostic results. This advancement could significantly accelerate the adoption of self-testing and home diagnostic applications.

### 2.19. Biomimetic Chiral Helical Nanoarchitectonics

**Figure 11:**
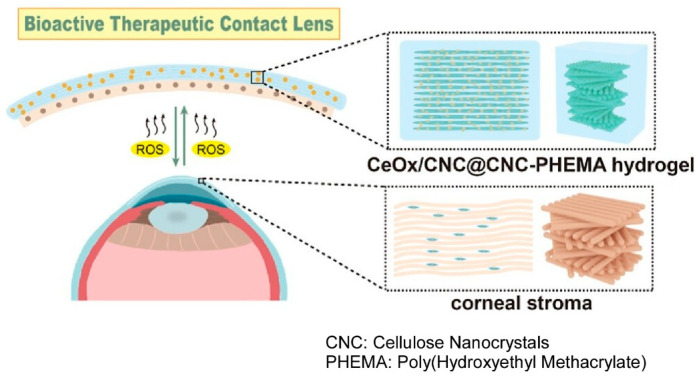
Fabrication of bioactive therapeutic contact lens mimicking the layered helical structure of the natural cornea upon integrating cellulose nanocrystals into poly(hydroxyethyl methacrylate) and forming CeO_x_ on the surface of the cellulose nanocrystals. Reprinted with permission from [311]. Copyright 2025 American Chemical Society.

Nanoarchitectonics-based material design and creation can also be applied to more practical uses. One example is the development of therapeutic contact lenses. The challenge in developing such lenses is to achieve rapid repair of severe corneal epithelial defects while regulating the oxidative stress environment. In the paper, ‘Bioactive therapeutic contact lens triggered by biomimetic chiral helical nanoarchitectonics for rapid corneal repair’, Zhu, Zhao and coworkers designed a bioactive therapeutic contact lens that mimics the layered helical structure of the natural cornea [311]. This was achieved by integrating cellulose nanocrystals into poly(hydroxyethyl methacrylate) and forming CeO_x_ on the surface of the cellulose nanocrystals (Figure 11). This hydrogel has a chiral helical structure that controls the microenvironment, and the nanoscale CeO_x_ on the surface of the cellulose nanocrystals acts as a reactive oxygen species (ROS) scavenger. This makes the hydrogel a bioactive therapeutic contact lens that promotes rapid corneal repair. Experiments using in vitro and in vivo corneal injury models revealed that this hydrogel has antioxidant, anti-inflammatory and anti-angiogenic properties, enables rapid migration of corneal epithelial cells and contributes to the regulation of the ocular surface microenvironment. This material, which can stimulate cell growth and migration, is expected to be useful in future corneal tissue engineering applications.

### 2.20. Nanoarchitectonics of Cello-Oligosaccharide

**Figure 12:**
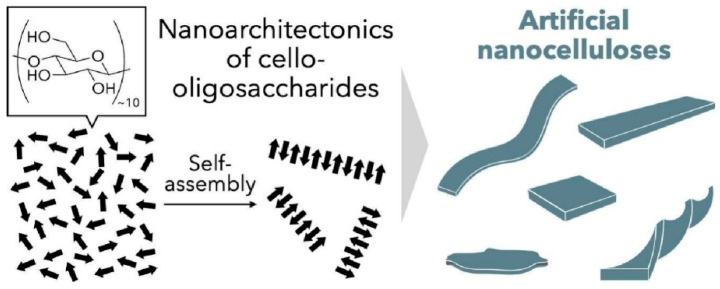
Nanoarchitectonics of artificial nanocellulose upon the preparation and self-assembly of cello-oligosaccharides. Reproduced under terms of the CC-BY license [312]. Copyright 2025 Elsevier.

Biomolecules, including cellulose, are influential components in nanoarchitectonics research. Recent advances in the nanoarchitectonics of low-molecular-weight cellulose, i.e., cello-oligosaccharides, have paved the way for the development of artificial nanocellulose. Nanocelluloses composed of cello-oligosaccharides synthesized by enzymatic oligomerization or solid-phase glycan synthesis are known as synthetic nanocelluloses. These nanostructures are constructed abiotically at the molecular level in a bottom-up manner. By adjusting the assembly process and molecular design, the molecular orientation, nanomorphology and surface functionality of artificial nanocellulose can be controlled. In the review paper, ‘Nanoarchitectonics of cello-oligosaccharides: a route toward artificial nanocelluloses’, Hata and Serizawa outline the latest research on artificial nanocellulose, from the preparation and self-assembly of cello-oligosaccharides to their potential applications. In contrast to naturally derived nanocellulose, artificial nanocellulose is constructed from the bottom up at the molecular level (Figure 12) [312]. The unique properties of engineered nanocellulose include diverse nano-morphologies, ease of surface functionalization, brittleness, an abundance of terminal glucose residues and the high chemical purity of synthetic nanocellulose. Cellulose hydrolysis can produce cello-oligosaccharides from abundant biomass. Further research into the hydrolysis mechanism and product fractionation will enable the production of chemically pure cello-oligosaccharides with controlled molecular structures. These properties distinguish engineered nanocellulose from naturally occurring nanocellulose in terms of applications. Engineered nanocellulose, composed of cello-oligosaccharides, is an emerging nanomaterial with diverse applications in materials science.

### 2.21. DNA Nanoarchitectonics

DNA is a powerful biomaterial for the rational design and functional enhancement of nanostructures. Deploying DNA nanoarchitectures at substrate interfaces offers unique advantages that could lead to improved surface properties relevant to biosensing, nanotechnology, materials science and cell biology. In a Perspective article titled ‘DNA nanoarchitectonics: assembled DNA at interfaces’, Howarka outlines the advantages and challenges of using assembled DNA as nanoscale building blocks in interfacial layers [313]. He outlines three specific applications: homogeneous, dense surface coatings; bottom-up nanopatterning; and 3D nanoparticle lattices. The first of these is homogeneous films focused on biomolecular recognition, the second is 2D nanopatterns fabricated by bottom-up methods and optionally combined with top-down nanofabrication, and the third is 3D nanoparticle superlattices. In conclusion, this review emphasizes the potential for future research into interfacial DNA nanostructures. DNA nanotechnology at interfaces is a highly interdisciplinary field spanning the nanoscale to the microscale, with numerous potential applications in the materials and life sciences.

### 2.22. Supramolecular Peptide Nanoarchitectonics

Peptides, which can be designed and synthesized in various structures, are also a popular subject in nanoarchitectonics research. In the paper, ‘Dual-integrin-targeted supramolecular peptide nanoarchitectonics for enhanced hepatic delivery and antifibrotic therapy’, Huang, Yan, Zou and coworkers reported the design of self-assembling peptides with dual integrin-binding motifs for α5β1 and αvβ3 [314]. Incorporating integrin-binding peptides into self-assembling building blocks is essential for developing targeted nanoarchitectonics. By incorporating dual integrin-binding peptides, multifunctionality was achieved. Using these peptides, this approach demonstrated supramolecular peptide nanoarchitectonics for enhanced liver delivery and antifibrotic therapy. Supramolecular nanoarchitectonics offer high drug loading capacity, favorable structural stability, efficient targeting and excellent biocompatibility, opening up new avenues for the treatment of liver fibrosis. As integrins are also overexpressed in other fibrotic organs and tumors, the design principles of this study can be applied to the development of peptide nanoarchitectonics for enhanced targeting and therapeutic efficacy.

### 2.23. Hybrid Nanoarchitectonics with Bacterial Component

Multifunctional graphene oxide shows promise for use in biomedical applications. Medically effective materials have been fabricated using graphene oxide nanoarchitectonics. In the paper, ‘Hybrid nanoarchitectonics with bacterial component-integrated graphene oxide for cancer photo-thermo-chemo-immunotherapy’, Chintalapati and Miyako developed a bioinspired approach using the cellular components of tumor-isolated Cutibacterium acnes to enhance the water dispersibility, drug-loading capacity, photothermal conversion efficiency and therapeutic immunogenicity of graphene oxide [315]. This approach enables camptothecin to target tumors locally with greater effectiveness, while the photothermal effect of graphene oxide promotes tumor heating. At the same time, Cutibacterium acnes components effectively activate the immune system, showing promise for treating advanced cancers. This work improves patient outcomes and paves the way for immune-boosting and nanohybrid-based therapies in oncology. Furthermore, this study contributes to the growing interest in nanomedicine and immunotherapy as effective strategies. Functional graphene oxide nanohybrids can be spatiotemporally activated by biologically penetrating near-infrared laser and anticancer drugs, resulting in effective tumor regression in mice. These findings provide a strong foundation for further research toward the optimization and clinical application of this nanomedicine strategy.

### 2.24. Cell Membrane-Camouflaged Nanoarchitectonics

Nanoarchitectonics techniques can be used to construct materials with diverse architectures, including biomimetic nanoparticles. In the study titled ‘Cell membrane-camouflaged nanoarchitectonics of photosensitizer nanoparticles for enhanced phototherapy in surgery’, Zhao, Zhang, Li and coworkers reported on the development of cancer membrane-mimetic nanoparticles composed of chlorin e6 (Ce6) and chlorambucil (CRB) [316]. The hydrophobic chemotherapeutic drug CRB was used to control Ce6 assembly through hydrogen bonding and π-π stacking interactions. The diameter of the nanoparticles could be adjusted from 100 nm to 2 µm by altering the reactant-to-solvent ratio. Ce6@CRB nanoparticles exhibited excellent photothermal conversion efficiency, twice that of free Ce6. Furthermore, Ce6@CRB nanoparticles could generate singlet oxygen more stably than free Ce6, thereby reducing oxygen dependence. Furthermore, coating the 4T1 cancer membrane on the Ce6@CRB nanoparticle surface conferred homologous targeting ability, improving Ce6 utilization. Additionally, combining photodynamic therapy with photothermal therapy effectively activates the immune system in vivo. Combining phototherapy with surgical resection minimizes the wound area as much as possible, optimizing both oncological safety and aesthetics. Interestingly, this treatment involves surgical resection after phototherapy, which effectively reduces the wound area. This research provides an effective method of tumor removal and is expected to be applied to clinical treatment from a patient-centered and humane perspective.

### 2.25. Liposomal-Based Nanoarchitectonics

**Figure 13:**
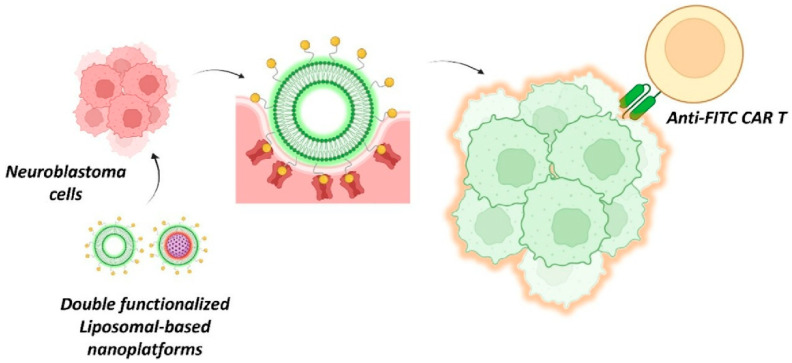
Liposomal-based nanoarchitectonics for targeting CAR T cells to neuroblastoma cells using nanometric bispecific T cell engagers, in which the outer lipid bilayer is decorated with strong affinity for the norepinephrine transporter as well as for fluorescein, which is recognized by anti-FITC CAR-T cells. Reprinted with permission from [317]. Copyright 2025 American Chemical Society.

Genetically engineered lymphocytes incorporating chimeric antigen receptors (CAR T cells) have enhanced their natural ability to seek out and destroy tumor cells, providing an effective and safe strategy for tumor eradication. However, their efficacy is generally limited. In their study, ‘Liposomal-based nanoarchitectonics as bispecific T cell engagers in neuroblastoma therapy’, Baeza et al. demonstrated a strategy for targeting CAR T cells to neuroblastoma cells using nanometric bispecific T cell engagers (Figure 13) [317]. In this design, the outer lipid bilayer is decorated with para-aminobenzyl guanidine, which exhibits strong affinity for the norepinephrine transporter that is overexpressed by neuroblastoma cells, as well as for fluorescein, which is recognized by anti-FITC CAR-T cells. The silica core can access the interior space by fusing with the neuroblastoma cell membrane via the protocell lipid membrane. These nanometric bispecific T-cell engagers combine the ability to label neuroblastoma cells with the capacity to deliver therapeutic agents to tumor cells, providing a novel approach to enhancing the efficacy of CAR T-cell therapy. These nano-metric bispecific T-cell engagers are modular and easily tunable, allowing them to be adapted to a wide range of malignancies and for use in treating other solid tumors. This approach can be easily adapted for the treatment of various solid malignancies and is expected to pave the way for the development of a new family of CAR T enhancers.

### 2.26. Nanoarchitectonics to Entrap Living Cell

**Figure 14:**
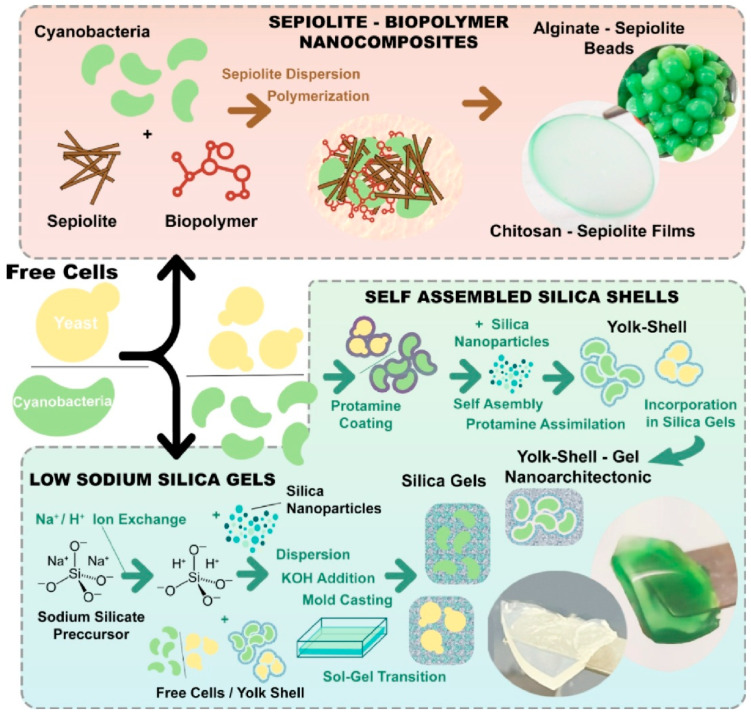
Nanoarchitectonics techniques to fabricate biohybrid materials from the bottom up for the encapsulation of living cells, in which unicellular microorganisms, namely cyanobacteria and yeast cells, were immobilized in silica- and silicate-based matrices organized as nanostructured materials. Reproduced under terms of the CC-BY license [318]. Copyright 2025 Beilstein Institute.

Research in nanoarchitectonics has also reported on the use of cells themselves as building materials. In the paper, ‘Nanoarchitectonics to entrap living cells in silica-based systems: encapsulations with yolk–shell and sepiolite nanomaterials’, Ruiz-Hitzky and coworkers used nanoarchitectonics techniques to fabricate biohybrid materials from the bottom up for the encapsulation of living cells (Figure 14) [318]. Unicellular microorganisms, namely cyanobacteria and yeast cells, were immobilized in silica- and silicate-based matrices organized as nanostructured materials. For instance, bio-nanocomposite-based matrices comprising a combination of chitosan and alginate with sepiolite clay mineral, molded into films, beads, or foams, were successfully employed to immobilize cyanobacteria. The silica shell microstructure was found to reduce cell–cell contact. The inorganic matrix enhanced cell viability and maintained bioactivity. Therefore, the efficiency of encapsulation was improved compared to methods involving direct contact of cells within a silica matrix. The encapsulated yeast produced ethanol over several days, suggesting the potential usefulness of this method as a biocatalyst. This nanoarchitectonics approach could pave the way for novel biohybrid systems with a wide range of applications, from preserving living cells to developing novel whole-cell bio-inorganic catalytic materials.

### 2.27. Cell-in-Catalytic-Shell Nanoarchitectonics

Nanoarchitectonics encompasses the creation of cell-shell biohybrid structures, which are formed by encapsulating individual living cells in exogenous materials. These structures have emerged as an exciting new class of functional entities for engineering biomaterials, with properties that extend beyond biochemical modification. In the paper, ‘Cell-in-catalytic-shell nanoarchitectonics: catalytic empowerment of individual living cells by single-cell nanoencapsulation’, Choi et al. presented a simple and flexible approach to imparting exogenous catalytic capabilities to living cells by nano-encapsulating them in a supramolecular metal–organic complex of Fe^3+^ and benzene-1,3,5-tricarboxylic acid [319]. A series of enzymes were embedded in the nanoshells in situ without compromising their catalytic activity. The supramolecular self-assembly of Fe^3+^ and benzene-1,3,5-tricarboxylic acid generated surface-active species in situ, leading to the spontaneous formation of nanofilms and shells on almost any substrate, including living cells, while simultaneously embedding the enzymes. These nanoshells enhance the catalytic efficiency of multienzyme cascade reactions by trapping reactive intermediates within their internal cavities. Nanoencapsulated cells can acquire exogenous biochemical functions, such as converting toxic chemicals into nutrients. For instance, nanoencapsulated cells can enzymatically degrade lethal octyl-β-D-glucopyranoside to D-glucose and possess autonomous cytoprotective functions. This approach could provide a valuable molecular toolkit for combining biological cells with non-natural materials to design next-generation cellular hybrid systems, which show great promise in biomedical and nanobiomedical applications.

### 2.28. Machine Learning in Nanoarchitectonics

The final topic is the application of artificial intelligence (AI) techniques, such as machine learning, in nanoarchitectonics. There is a strong and profound connection between nanoarchitectonics and machine learning. Mathematics has historically played a pivotal role in materials synthesis, influencing numerous fields including nanoscience and nanoarchitectonics. In the review paper, ‘Machine learning in nanoarchitectonics’, Skirtach et al. analyze the use of artificial intelligence, machine learning and deep learning in the discovery, prediction, optimization, characterization and imaging of nanoarchitectonics [320]. Machine learning is commonly used in atomic and molecular science, nanotechnology of colloids and nanofilms, and micro- and macro-engineering, for example. Machine learning is particularly important in nanotechnology for colloids and nanofilms, as nanofabricated structures often do not match predicted nanodesigns. This review analyses machine learning approaches, including models and algorithms, and attempts to link them to material properties at various scales. This research trend is expected to ultimately lead to the autonomous optimization of material properties at different scales. Autonomous synthesis involves automating the manufacturing process of materials such as nanoparticles by using machine learning algorithms and advanced robotics to streamline and optimize synthesis conditions. Using machine learning models to predict outcomes based on a set of input parameters and experimental conditions enables the rapid identification of optimal synthesis protocols. For instance, integrating machine learning with automated synthesis platforms can considerably reduce the time and resources needed to produce functional nanomaterials, such as nanoparticles. Autonomous synthesis requires the development of technologies such as machine learning, laboratory automation and robotics, as well as remote connectivity, virtual reality tools, and autonomous driving based on these technologies. Such approaches hold great promise in advancing the field of nanoarchitectonics.

## 3. Future Perspectives

This review paper highlights the broad scope of applications of the new concept of nanoarchitectonics, selecting and discussing recent papers that contain the term ‘nanoarchitectonics’ in their titles. Rather than attempting to organize them systematically, the selection of papers is broad and diverse, reflecting the wide range of research topics covered. Topics include highly precise structural control at the atomic and molecular level, such as controlling the number of dopant atoms in solid electrolytes, transforming the framework of carbon atoms in carbon materials, developing single-atom catalysts, and creating nanorobots and microrobots. Various nanomaterials are also included. Examples of research include functional nanoparticles, nanotubular materials, 2D-organic nanosheets and MXene nanosheets. Applications of nanostructured functional materials include nanosheet assemblies, nitrogen-doped carbon, nanoporous and mesoporous materials, and nanozymes. Furthermore, polymeric materials engineered through organic chemistry, covalent organic frameworks and vesicle structures constructed from synthetic polymers are also the focus of nanoarchitectonics research. Research is also being conducted into supramolecular chemistry and controlled structures created by various methods, such as chirality- and topology-controlled structures, chiral helices, Langmuir monolayers, LB films, and LbL assembly. The nanoarchitectonics of biological materials is also a widely pursued field of study. Nanocellulose, DNA, peptides and bacterial cell components are used in this field. Bio-related nanoarchitectonics also involves creating more complex structures, such as biomimetic nanoparticles and materials that mimic the lipid membranes of protocells. Research is also being conducted into the organization of living cells themselves and the encapsulation of living cells with exogenous substances. Thus, nanoarchitectonics targets a very wide range of functional material systems, from atomic and molecular control to cellular structure. The examples presented here are only a small selection, and the scope of nanoarchitectonics research is extremely broad. Nanoarchitectonics represents a conceptual framework for creating materials at all levels and can be likened to a method for everything in materials science.

In this review, examples are selected with term of nanoarchitectonics in the title. However, many other research approaches can contribute to nanoarchitectonics even without using this terminology. For example, the pulsed laser ablation method and its variations are one of the most flexible tools for producing complex nanostructures, including core–shell structures, hybrids, and alloys [321,322]. Application of external inputs by lasers can provide huge variations of structure modulations in dimensional, phase, and composition control through selecting laser parameters, medium type, and processing sequence, which all allow for fine-tuning the functionality of the resulting structures with optical, magnetic, and catalytic properties. Such combined strategies contribute nanoarchitectonics strategies. Not limited to these cases, many existing methods can share their importance with nanoarchitectonics.

Here, I would like to focus on the final topic: the application of artificial intelligence technology to nanoarchitectonics. This example demonstrates how machine learning and automated synthesis techniques can contribute to nanoarchitectonics research on functional nanoparticles. Introducing AI technology into nanoarchitectonics research will be a significant milestone. Recent research papers have highlighted the contributions of machine learning methodologies to functional materials and various scientific fields [323,324,325,326,327,328]. There is also a growing trend in nanoarchitectonics research towards incorporating AI-related technologies [329,330,331]. AI technology can be applied to nanoarchitectonics research in various ways, including optimizing synthesis conditions and using vast amounts of information to design unprecedented functional materials. As this review shows, the targets of nanoarchitectonics research are extremely diverse. Using AI technology to create functional materials that cannot be imagined based on experience or rules by incorporating these data sets is a very attractive prospect. Highly functional systems, such as those found in living organisms, are coordinated and harmonized in such a way that they exhibit extremely high functionality and flexible adaptability [332,333,334]. We must establish a methodology for creating such functional material systems. Rather than developing materials specialized in a particular field, we need a development approach similar to that of biological functional systems, incorporating a variety of elements. In such an approach, it will be important to introduce AI technology capable of handling large amounts of information. In addition, strategies with machine learning in nanoarchitectonics for functional materials such as nanoparticles can lead to condition optimization and automatic syntheses. Such approaches enable us to evaluate many possibilities without tedious experimental trials. Introduction of artificial intelligence into nanoarchitectonics approaches has a huge future potential in a wide range of target materials and functions.

Developing technology that can universally create materials with unexpected functions could represent the final frontier of materials science. Nanoarchitectonics will play a significant part in achieving this final frontier in materials science, although several issues including scalability, ethical issues, and reproducibility have to be further considered. So, what kind of functional materials should we ultimately aim for? They should be highly functional systems, similar to those found in biological systems. Photosynthesis, mate-rial transformation, and signal transduction operate with exceptional efficiency and selectivity. Moreover, these processes occur in an aqueous environment at room temperature and pressure. These highly functional material systems are exactly what we should strive for. In biological systems, many functional units work in harmony and are interconnected. These functional systems are ingeniously constructed within a single system. Developing such systems is the ultimate goal of nanoarchitectonics. Biological systems have developed these systems over billions of years of evolution. Nanoarchitectonics is based on a comprehensive concept that allows for the use of any and all functional materials. With the help of AI, the goal of human science and technology is to achieve such a feat within a few decades.
